# Putting a Finger on Numerical Development – Reviewing the Contributions of Kindergarten Finger Gnosis and Fine Motor Skills to Numerical Abilities

**DOI:** 10.3389/fpsyg.2020.01012

**Published:** 2020-05-26

**Authors:** Roberta Barrocas, Stephanie Roesch, Caterina Gawrilow, Korbinian Moeller

**Affiliations:** ^1^Leibniz-Institut fuer Wissensmedien, Tuebingen, Germany; ^2^Department of Psychology, LEAD Graduate School & Research Network, Eberhard Karls University Tuebingen, Tuebingen, Germany; ^3^Centre for Mathematical Cognition, Loughborough University, Loughborough, United Kingdom

**Keywords:** finger gnosis, fine motor skills, finger counting, numerical development, embodied numerosity, finger-based numerical strategies, mathematics achievement

## Abstract

The well-documented association between fingers and numbers is not only based on the observation that most children use their fingers for counting and initial calculation, but also on extensive behavioral and neuro-functional evidence. In this article, we critically review developmental studies evaluating the association between finger sensorimotor skills (i.e., finger gnosis and fine motor skills) and numerical abilities. In sum, reviewed studies were found to provide evidential value and indicated that both finger gnosis *and* fine motor skills predict measures of counting, number system knowledge, number magnitude processing, and calculation ability. Therefore, specific and unique contributions of both finger gnosis and fine motor skills to the development of numerical skills seem to be substantiated. Through critical consideration of the reviewed evidence, we suggest that the association of finger gnosis and fine motor skills with numerical abilities may emerge from a combination of functional and redeployment mechanisms, in which the early use of finger-based numerical strategies during childhood might be the developmental process by which number representations become intertwined with the finger sensorimotor system, which carries an innate predisposition for said association to unfold. Further research is nonetheless necessary to clarify the causal mechanisms underlying this association.

## Introduction

Fingers and numbers seem to be inextricably associated. Almost all children across different cultures use their fingers for counting and initial calculation (e.g., Carpenter and Moser, 1982; Fuson and Hall, 1983; Fuson, 1988; Butterworth, 1999), and most cultures seem to develop specific finger-based counting strategies and systems (e.g., Butterworth, 1999; Ifrah, 2000; Bender and Beller, 2012). Even blind children use their fingers for counting and displaying numerical magnitudes (Crollen et al., 2011). Moreover, a growing body of literature dedicates itself to examining this association, both on a behavioral and neuro-functional level. Perhaps, one of the most intriguing sets of evidence among these is the well-documented association between finger motor and sensory abilities – that is, the capability of differentially moving and mentally representing one’s fingers [henceforth referred to as fine motor skills (FMS) and finger gnosis, respectively] and basic numerical abilities in early childhood (e.g., Noël, 2005; Grissmer et al., 2010). In this context, one study even found that training of finger gnosis improved numerical performance in first graders (Gracia-Bafalluy and Noël, 2008; but see Fischer, 2010). Although many studies seem to substantiate the existence of this association, its driving mechanisms remain largely unexplained. Disclosing these mechanisms requires a critical evaluation of the existing evidence on the association of fingers and numbers in preschool age. In this article, we briefly review developmental studies evaluating the association of finger gnosis and FMS with basic numerical abilities in preschool age.

### Early Numerical Development

The ability to reason with numbers is critical for individual life and career prospects (Dowker, 2005; Duncan et al., 2007; Butterworth et al., 2011; Ritchie and Bates, 2013). Importantly, however, the foundations of numerical development are laid long before children get in contact with formal mathematical instruction (e.g., Siegler and Braithwaite, 2017; for a review). Instead, they begin to unfold in early childhood when children first learn how to count and understand the meaning of number magnitude. These basic, early numerical abilities constitute building blocks for more complex arithmetic and mathematical competences in the future (e.g., Jordan et al., 2009).

Given their importance, it is unsurprising that the development of children’s basic numerical abilities has prompted the interest of researchers across different disciplines. The study of children’s early understanding of number can be traced back to Piaget (1952) constructivist theory, in which he advanced the concept of *equinumerosity* (i.e., the comprehension that the cardinality of two sets of objects are equivalent only when their components can be paired with each other in one-to-one correspondence) as the cornerstone of numerical understanding.

Expanding on Piaget’s theory, cognitive psychologists Gelman and Gallistel (1978) introduced an influential view on numerical development, which stated that the act of counting following designated counting principles (i.e., stable order, one-to-one correspondence, and cardinality) is already in itself is an indication of children’s ability to represent number. The authors further argue that the acquisition of counting requires the construction of a bi-directional mapping system of innate preverbal, analog magnitudes onto their corresponding symbolic representations (Gallistel and Gelman, 1992). This rationale is also echoed by more recent theories of early numerical development which accentuate the importance of acquiring the ability to map non-symbolic onto symbolic representations of number (e.g., Siegler and Lortie-Forgues, 2014). Furthermore, as children take their initial steps into a numerate world, they learn how to represent non-symbolic magnitudes with increasing precision, acquire number concepts and number words, counting procedures, and cardinality knowledge (Geary, 2007).

However, also authors in the field of mathematics education elaborated on children’s acquisition of counting skills as an important milestone preceding their understanding of number. For instance, Steffe et al. (1982) described three types of counting in which pre-numerical children operate with either perceptual, figural, and/or motor unit items. These procedures differ in their degree of reliance on immediate perception of the to-be-counted objects and are claimed to give rise to different ways of mentally operating on numbers for problem-solving, with counting motor unit items (i.e., by moving fingers or other body parts) being the type with least reliance on the material presence of counting units. Through the acknowledgment of finger use as a sophisticated, effective means of mentally manipulating numerical information, Steffe et al. (1982), alongside Fuson (1982) and later followed up by Brissiaud (1992), considered finger-based strategies in a theoretical framework of early numerical development within the mathematics education literature.

### Fingers and Numbers

The importance of fingers for the development of early numerical abilities is reflected in Butterworth’s (1999) claim that numerical representations are partially supported by FMS and finger gnosis. Moreover, finger counting has been argued to be a prototypical instance of embodied cognition (Fischer and Brugger, 2011). This means that numerical representations, once thought to be purely abstract, seem to be rooted in early sensorimotor experiences of finger counting (Moeller et al., 2012), which are assumed to leave a lasting trace on adult number processing in turn (Di Luca and Pesenti, 2011). The embodiment of numerical concepts and processes has been demonstrated by numerous studies dedicated to evaluating sensory and motor biases in adult numerical cognition (e.g., Fischer, 2003; Andres et al., 2004; Badets et al., 2010; Sixtus et al., 2017), as well as studies reporting influences of finger-based numerical representations on number processing. For instance, Domahs et al. (2008) found that second graders tend to commit specific split-five errors (i.e., erroneous answers deviating by ±5, and thus by one hand, from the correct result) when solving mental arithmetic problems. Furthermore, Domahs et al. (2010) reported significant effects of counting habits on magnitude processing of Arabic digits, and finger movement has been found to interfere with mental calculation even in adults (Michaux et al., 2013; Soylu and Newman, 2016).

Beyond this behavioral evidence, results from neurophysiological studies provide converging evidence for an association of fingers and numbers already at the neural level. In this context, numerous functional neuroimaging studies indicated overlapping activation of cortical networks for number processing and finger movement starting from childhood (e.g., Simon et al., 2002, 2004; Krinzinger et al., 2011; Tschentscher et al., 2012; Berteletti and Booth, 2015) – albeit with slight developmental differences. For instance, Kaufmann et al. (2008) observed significantly higher activation of areas responsible for finger-related movements in children than in adults when processing non-symbolic numerosities in addition to areas typically found to be involved in number magnitude processing (i.e., the intraparietal sulcus). Moreover, Rusconi et al. (2005) expanded on these neuroimaging results by applying transcranial magnetic stimulation to the left angular gyrus. They observed this to disrupt both finger gnosis and number processing in adults, which substantiates the assumption of a functional link between the neural representation of fingers and numbers. This idea is further corroborated by electrophysiological evidence indicating increased corticospinal excitability of right-hand muscles on a parity judgment task with small numerals (i.e., 1–4) in participants who started counting on their right thumbs from one to five (Andres et al., 2007). These results suggest that hand motor circuits were activated during non-symbolic number processing in adults (Andres et al., 2007), and this effect seems to be modulated by individual differences in finger counting routines (Sato et al., 2007). Taken together, these findings were argued to be indicative of intertwined cortical representations for numbers and fingers, which may be reminiscent of embodied numerical strategies in childhood.

**FIGURE 1:**
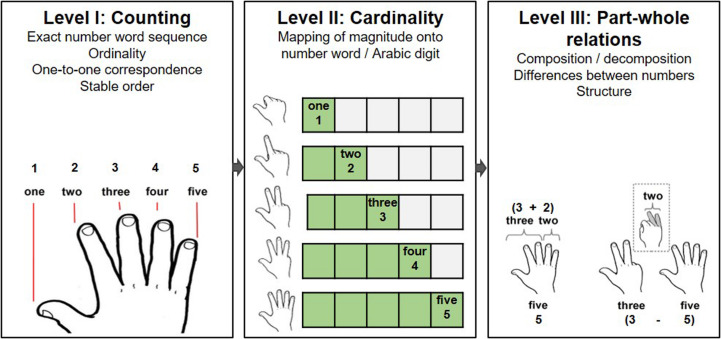
Schematic depiction of how fingers may support acquisition of basic numerical abilities according to influential theories of early numerical development (Gelman and Gallistel, 1978; Brissiaud, 1992; Geary, 2007; Krajewski and Schneider, 2009). Adapted with permission from Roesch and Moeller (2015).

There are many ways in which the use of fingers may functionally support the acquisition of basic numerical abilities (and thereby engender the embodiment of numerical representations). Considering the three levels of basic numerical development suggested by the model of Krajewski and Schneider (2009) (see Figure 1), the act of *counting* on one’s fingers may help children get acquainted with the one-to-one correspondence principle (Brissiaud, 1992), as well as convey the counting principles of stable order and ordinality (Crollen et al., 2011). Furthermore, the finger patterns depicting numerical quantities may facilitate the acquisition of the *cardinality* principle (Brissiaud, 1992) and advance the comprehension of *part-whole relations* (Gattegno, 1974; Brissiaud, 1992; Krajewski and Schneider, 2009). Additionally, fingers may also help convey a sense of structure (Björklund et al., 2019) and hint at the base-10 structure of the number system. As previously pointed out, these abilities are consensually regarded as fundamental for the development of mature numerical reasoning within both the domain of mathematics education and (cognitive) psychology (see Figure 1).

Nevertheless, as noted by Moeller et al. (2011), the use of fingers in support of numerical learning has been subject of controversy among researchers in the fields of (neuro-cognitive) psychology and mathematics education. The question of whether fingers constitute a scaffold or rather a hinderance for numerical development resides in the epicenter of these discussions. Recently, the notion that finger usage is a strategy adopted mostly by children with mathematical difficulties (e.g., Neuman, 1987) or cognitively low-performing children was challenged by evidence showing that 6-year-old children with high working memory capacity were more likely to use finger-based strategies than children with low working memory capacity – with these strategies also leading to better arithmetic performance (Dupont-Boime and Thevenot, 2018). In conjunction with the body of work supporting the perspective of embodied numerosity, this finding may hint toward the need to shift attention from fingers as putative cognitive crutches to seek a clearer understanding of individual differences in the use of finger-based numerical strategies, as well as likely scenarios in which finger use may be less or more effective in dealing with numerical information.

Crucially, the successful use of finger-based strategies depends not only on the intuition that fingers may be used as tools for representing and computing numerical quantities, but also largely on the ability to perform the intricate, fine-grained movements required for counting and producing specific finger postures. In support of this view, several studies documented an association between FMS and finger gnosis (i.e., the ability to move and mentally represent one’s fingers) and performance in basic numerical abilities in early childhood (e.g., Noël, 2005; Grissmer et al., 2010). Recently, Soylu et al. (2018) provided an interesting review focusing largely on the role of finger gnosis for early mathematics development and not particularly considering FMS. Therefore, considering the influences of basic finger motor in addition to sensory finger abilities on the development of early numerical abilities may be a promising direction for better understanding the almost universal appeal of fingers for supporting learning and processing of numerical content.

In particular, the ability to mentally represent, discriminate between, display and locate one’s fingers is most commonly termed finger gnosis (e.g., Penner-Wilger et al., 2007; Reeve and Humberstone, 2011). Finger gnosis has been claimed to be one of the fundamental competences supporting the development of numerical skills (Butterworth, 1999), and associations between finger gnosis and numeracy have been observed in both typical and clinical populations (e.g., developmental Gerstmann syndrome, Gerstmann, 1940; Benson and Geschwind, 1970; Suresh and Sebastian, 2000). Beyond finger gnosis, FMS have also been argued to support numerical processing and development (Butterworth, 1999). The association between academic achievement and FMS, that is “control and coordination of the distal musculature of the hands and fingers” (Bruininks and Bruininks, 2005), was the subject of numerous studies over the last decades (e.g., Keogh and Smith, 1967). Historically, FMS have also been termed visual-motor integration, perceptual-motor ability or psychomotor skills. The association between FMS and numerical skills has been observed in both typically developing children (e.g., Grissmer et al., 2010) as well as in clinical populations with motor impairments such as cerebral palsy (e.g., van Rooijen et al., 2012, 2016), developmental coordination disorder (e.g., Holsti et al., 2002; Pieters et al., 2012, 2015; Gomez et al., 2015) and spina bifida myelomeningocele (e.g., Barnes et al., 2005, 2011; Raghubar et al., 2015). The origin of this association has been assumed to rely on either simultaneous maturation, subordination of both to general intelligence (Luo et al., 2007), more stimulating home environments corroborating both FMS and cognitive development (McPhillips and Jordan-Black, 2007; Suggate et al., 2017b), a functionally or culturally driven connection (Butterworth, 1999; Fischer et al., 2017), or FMS building the fundamental basis of cognitive development, which has been claimed to be embodied by nature (e.g., Lakoff and Núñez, 2000; Thelen, 2000). The emergence of the intriguing association between fingers and numbers can be interpreted under the light of different explanations (Penner-Wilger and Anderson, 2013): first, according to the *functionalist* proposition (Butterworth, 1999), fingers and numbers become associated through early developmental experiences of using fingers for counting and initial calculation. In this line of thought, the use of fingers in support of early numerical reasoning during childhood is the driving mechanism of the association of numerical abilities with finger sensorimotor skills (i.e., finger gnosis and FMS). Alternatively, a second explanation to these findings is that both finger and number representations recruit a common neural circuitry. According to the so-called *massive redeployment* view (Anderson, 2010; Penner-Wilger and Anderson, 2013), some of the neural circuits originally involved in finger representation may have been exapted or re-used through evolutionary mechanisms for supporting numerical cognition.

The key difference between these different accounts on the observed association of fingers and numbers lies in the relative weight attributed to the neurofunctional aspects of this association and the direction of its causality: while the functionalist hypothesis suggests that fingers and numbers may become associated on a neural level through the systematic experience of using fingers in the course of early numerical development, the massive redeployment hypothesis posits that the pre-existence of a shared neural substrate for fingers and numbers drives the use of fingers for numerical reasoning. Despite proposing diametrically different causal explanations, both functionalist and massive redeployment propositions are well-accepted within the literature and seem to gather similar degrees of support from different authors without a clear preponderance of one over the other. Therefore, to this day there is no consensus regarding the precipitating mechanisms of the association of fingers and numbers.

In this context, studies investigating the role of fingers for the acquisition of preschool numerical skills offer particularly relevant insights, as they may shed light on the association between fingers and numbers prior to the onset of functional strategies, that is, before (or around the time) children start using their fingers for counting and representing numerical magnitudes. A critical consideration of these studies’ contributions may be a promising direction to elucidate which causal mechanisms may be responsible for shaping this association, as well as help extricate functionalist and massive redeployment explanations of these findings.

In this article, we review developmental studies evaluating the association between fingers and numerical skills in typically developing preschool children. Drawing partially (but not exclusively) on Butterworth (1999) theoretical framework, we will specifically focus on research targeted at FMS and finger gnosis. After briefly elaborating on our search strategy and describing all thereby obtained studies, we will discuss how both variables relate to children’s numerical development, reflect on their constraints and suggest potential directions for future research. Finally, we discuss the scope and limitations of the two main explanatory propositions of these findings considering current neuro-functional evidence.

### Search Strategy and Inclusion Criteria

Studies were searched up to October 2019 in PsycARTICLES and PsycINFO. Search terms included “fingers,” “finger gnosis,” “finger gnosia,” “finger sense,” “fine motor skills,” “finger dexterity,” “finger tapping,” and “finger agility” in combination with the terms “numerical skills,” “numerical development,” “numerical cognition,” and “mathematics achievement,” filtering the results for the age group of preschool. The search produced 543 hits on PsycINFO and PsycArticles. Titles and abstracts of these studies were manually scanned for relevance. All peer-reviewed articles (published in journals or conference proceedings) focusing on the longitudinal and concurrent association between finger-related variables and the development of numerical skills in preschool age through the first school years were considered in this review. References from the relevant studies were further inspected for additional studies to be considered. Research articles focusing on clinical subgroups (e.g., children with cerebral palsy, van Rooijen et al., 2012), adults (Penner-Wilger et al., 2014), older school-aged children (e.g., Carlson et al., 2013) and published in languages other than English or German were not considered for the present review. This resulted in a final set of 20 studies considered in this review.

## Results

### Finger Gnosis and Numerical Abilities

In recent years, the impact of finger gnosis on typically developing preschoolers’ numerical abilities has been investigated following the idea that – if finger gnosis indeed constitutes a building block for the development of numerical abilities (e.g., Butterworth, 1999) – better finger gnosis should be associated with better numerical abilities.

**FIGURE 2:**
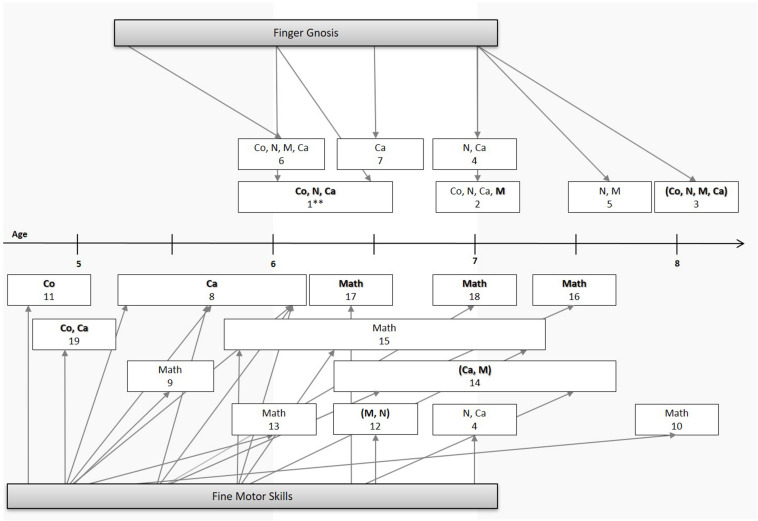
Overview of developmental studies on the influence of fine motor skills (below the line) and finger gnosis (above the line) on numerical skills and mathematics achievement. Co, counting; N, number knowledge; Ca, calculation; M, magnitude; Math, mathematics achievement. For simplification purposes, children’s mean ages upon assessment of independent and outcome variables were rounded up or down in intervals of 0.5 year ranging from age 5 to 8. Medium to large effect sizes are represented in bold typeface. Outcome variables composed of different numerical measures but expressed in one single score are given in brackets. **Study used a predictor variable based on a composite measure of sensory-motor skills. The studies are, in order: 1. Fayol et al. (1998), 2. Long et al. (2016), 3. Noël (2005), 4. Penner-Wilger et al. (2007), 5. Penner-Wilger et al. (2009), 6. Poltz et al. (2015), 7. Wasner et al. (2016), 8. Asakawa and Sugimura (2014), 9. Cameron et al. (2012), 10. Dinehart and Manfra (2013), 11. Fischer et al. (2017), 12. Gashaj et al. (2019), 13. Grissmer et al. (2010), 14. Kim et al. (2017), 15. Luo et al. (2007), 16. Pagani et al. (2010), 17. Pitchford et al. (2016), 18. Son and Meisels (2006), 19. Suggate et al. (2017a). The study of Gashaj et al. (2018) was not represented in the figure because the predicted association was indirect.

One of the first studies to investigate this claim found that a composite of sensory-motor measures including finger gnosis assessed in kindergarten was a better predictor of children’s numerical skills in first grade than a measure of their overall cognitive development (assessed by the “Draw-a-Person test,” Fayol et al., 1998). Similarly, Noël (2005) found that preschoolers’ finger gnosis significantly predicted their numerical skills, but not their reading ability, both concurrently and at the end of first grade (see Figure 2 for an illustration of these associations). Along with handwriting and block design, finger gnosis explained about 46% of variance of children’s later numerical skills (see Table 1 for more detailed information on the respective studies).

**TABLE 1:** Overview of studies examining the association between finger gnosis and numerical skills.

**References**	**Finger variable**	**Age (Y;M)**	**N**	**Task**	**Control variables**	**Numerical outcome variables^1^ (with reported effect sizes)**
Fayol et al. (1998)**^L^**	Neuropsychological Score^2^ (T1)	T1: 5;9 T2: 6;5	177	10 Trials; single and consecutive touch; pointing and label naming	Lozenge and human figure drawing test, age (T1)	**Co**: ***r* = 0.40** (T1) **N**: Number writing: *r* = 0.16 (T1); *r* = 0.27 (T2) Number sequence: ***r* = 0.42** (T1) **Ca:** Problem solving; ***r* = 0.40** (T1); ***r* = 0.45** (T2) All: ***r* = 0.50** (T1); ***r* = 0.46** (T2)
Long et al. (2016)**^C^**	Finger gnosis	7;1	197	50 Trials; single, consecutive and simultaneous touch; pointing	Age	**Co**: Dot counting*: r* = 0.10 **N**: Symbolic comparison*: r* = 0.06 **Ca**: *r* = 0.12 **M**: Non-symbolic comparison***: r* = 0.38**
Noël (2005)**^L^**	Finger gnosis (T1 and T2)	T1: 6;8 T2: 7;11	41	40 Trials; single, consecutive and simultaneous touch; pointing	Processing speed, hand preference, left–right orientation (T1), block design, handwriting (T2)	**Co, N, Ca, M**^3^: Numerical accuracy score ***r* = −0.48** (*FG T1*) ***r* = −0.36** (*FG T2*) Numerical speed score ***r* = −0.30** (*FG T1*) *r* = **−0**.01 (*FG T2*)
Penner-Wilger et al. (2007)**^C^**	Finger gnosis	6;10	146	20 Trials; simultaneous or consecutive touch; pointing	Gender, receptive vocabulary, processing speed	**N**: *r* = 0.27 **Ca**: *r* = 0.19
Penner-Wilger et al. (2009)**^L^**	Finger gnosis (T1)	T1: 6;10 T2: ∼7;10	100	20 Trials; simultaneous or consecutive touch; pointing	Gender, processing speed, receptive vocabulary (T1)	**N**: distance effect β = −0.35 (T2) **M**: number line estimation linearity β = 0.27 (T2)
Poltz et al. (2015)**^L^**	Finger gnosis (T1 and T2)	T1: 5;3 T2: 6;0	1,594	16 Trials; single and simultaneous touch; pointing	Nonverbal IQ, visual WM, selective attention, number skills T1)	*FG T1* **Co**: *r* = 0.26 (T1), *r* = 0.23 (T2) **N**: *r* = 0.26 (T1), *r* = 0.18 (T2) **Ca**: *r* = 0.33 (T1), *r* = 0.32 (T2) *FG T2* **Co**: *r* = 0.23 (T1), *r* = 0.20 (T2) **N**: *r* = 0.19 (T1), *r* = 0.15 (T2) **Ca**: *r* = 0.25 (T1), *r* = 0.30 (T2)
Wasner et al. (2016)**^C^**	Finger gnosis	6;5	321	21 Trials; single and consecutive touch; pointing and visual recognition	Age, gender, general cognitive ability, verbal and visual short-term memory, numerical precursor skills	**Ca**: Addition: *r* = 0.23, β = 0.14 Subtraction: *r* = 0.24, β = 0.13

*Medium to large effect sizes (Cohen, 1988) are displayed in bold typeface. Regression coefficients were not interpreted in terms of effect size. ∼Indicates an approximate mean age when exact means are not provided. Tests included in the *p*-curve analysis are underlined. L, longitudinal; C, cross-sectional. ^1^In order to facilitate comparisons between studies, we grouped the outcome variables into four different categories whenever possible (Wyschkon et al., 2015): Co, counting (i.e., forward, backward, from *x* to *y*), N, number (e.g., number reading, symbol-magnitude mapping, cardinality, and place value), Ca, calculation (e.g., addition and subtraction), M, magnitude (e.g., subitizing, size comparison, and magnitude judgment). Original outcome measure names as described in the studies are cited when different from category names.**^2^** Simultagnosia, finger gnosis, digital discrimination, and graphisthesia. ^3^Negative association because the study used a finger agnosia score.*

Building on these results, Penner-Wilger et al. (2007) found that finger gnosis assessed in first grade significantly predicted children’s concurrent calculation ability, although only indirectly through number system knowledge. Expanding on these findings longitudinally, Penner-Wilger et al. (2009) observed that children with better finger gnosis scores in first grade performed significantly better in a number magnitude comparison task 1 year later (see Figure 2). Additionally, finger gnosis significantly predicted linearity of estimates in a number line estimation task, claimed to reflect better numerical representations (Siegler and Booth, 2004).

**FIGURE 3:**
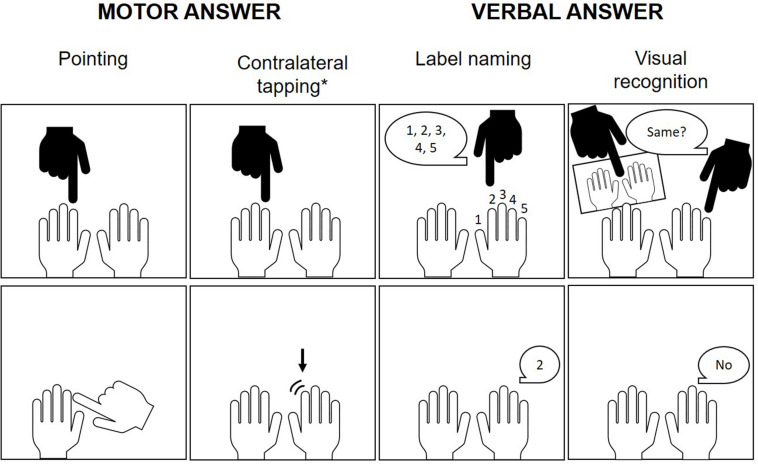
Response types of finger gnosis assessment paradigms in children. Beyond these differences, tasks also diverged in terms of whether or not the child’s hands were made visible after pointing to the finger to facilitate recognition, as well as in number of trials and whether fingers were pointed at individually, consecutively or simultaneously (or yet a combination of these stimulation modalities). *Although contralateral tapping was not present in any of the here reviewed studies, it was adopted by other authors investigating finger gnosis, such as Newman (2016).

Although these earlier studies seemed to corroborate an association between finger gnosis and numerical skills, it needs to be noted that they have important limitations which preclude a clear understanding of this association. While some lacked an analysis of the unique contribution of finger gnosis to numerical skills (Fayol et al., 1998; Noël, 2005), others used a finger gnosis task which had either a number processing or motor confound: for instance, Fayol et al. (1998) required participants to identify the touched finger by naming the number assigned by the experimenter to the respective finger, whereas Noël (2005); Penner-Wilger et al. (2007), and Long et al. (2016) asked children to point at the touched finger (see Figure 3 for more details on task specifics across studies; see also Guedin et al., 2018, for an alternative paradigm of finger gnosis measurement which may be more suited for younger children). Moreover, most studies did not control for the influence of other important predictors of numerical development such as general cognitive ability (Noël, 2005; Long et al., 2016) or numerical precursor skills (e.g., Fayol et al., 1998).

Attempting to tackle these issues, more recent studies found the predictive power of finger gnosis to be weaker than previously thought (Poltz et al., 2015; Long et al., 2016; Wasner et al., 2016). When controlling for numerical precursor skills, nonverbal IQ and other domain-general skills, Poltz et al. (2015) found that 5-year-olds’ finger gnosis was a unique predictor of their numerical skills at age six (see Figure 1), but accounted for only a small part of variance (about 2%). In line with this, Wasner et al. (2016) showed that finger gnosis was associated with first graders’ addition and subtraction performance, but again accounted for no more than 1–2% of variance when the influence of general cognitive ability, short term memory and numerical precursor skills (e.g., symbolic and non-symbolic magnitude comparison) was considered (for similar results see also Long et al., 2016).

Even though these findings seem to substantiate the hypothesis of a parallel development of finger gnosis and numerical abilities, it is important to note that the correlational design of two of these studies (Long et al., 2016; Wasner et al., 2016) does not permit causal interpretations of their results. For instance, in the study of Wasner et al. (2016), the fact that concurrently assessed finger gnosis accounted for little variance on numerical performance after controlling for numerical precursor skills does not rule out the possibility that these very basic numerical abilities being accounted for were acquired with assistance of finger-based strategies in earlier numerical development.

It is also important to acknowledge that, although most studies followed a common parameter for the assessment of finger gnosis (i.e., indicating the finger(s) stimulated by the experimenter; Baron, 2004), task specifics appear to be heterogeneous in what concerns number of trials, way of finger stimulation and response modality (see Figure 3 and Task column in Table 1), which may give rise to comparability issues. For instance, although most studies used a combination of trials comprising stimulation of one individual finger as well as consecutive or simultaneous stimulation of two fingers, some of them (Penner-Wilger et al., 2007, 2009) included only consecutive and simultaneous trials, which increases task difficulty. Moreover, while most experimental procedures allowed children to identify the touched finger(s) by means of visual guidance, one study (Long et al., 2016) required children to point to the touched fingers with their hands still out of sight. Additionally, as pointed out by Wasner et al. (2016), the internal consistency of finger gnosis tests was mostly weak throughout studies (see also Long et al., 2016 for a discussion of this point). Future studies should thus aim at establishing a standard way for measuring finger gnosis to avoid confounds and warrant comparability of research findings.

To evaluate the evidential value of the reviewed findings, we conducted a *p*-curve analysis (Simonsohn et al., 2014, 2015). This procedure allows for accounting for publication bias and provides an estimate of the true effect size associated with a given set of findings. For this analysis, we selected the significant coefficients based on the following criteria: (1) only one coefficient was chosen from each study (see Table 1 for disclosure); (2) in case coefficients were reported for both concurrent and longitudinal associations, preference was given to the longitudinal test; (3) in case more than one longitudinal coefficient was given, we opted for the association covering the age range and/or test interval closest to the one investigated by other studies; (4) for one study (Wasner et al., 2016) in which test results were provided for both addition and subtraction, we chose the result for addition due to consistency with other studies; (5) when tests from different studies were not independent (i.e., Penner-Wilger et al., 2007, 2009), only one of them was considered.

**FIGURE 4:**
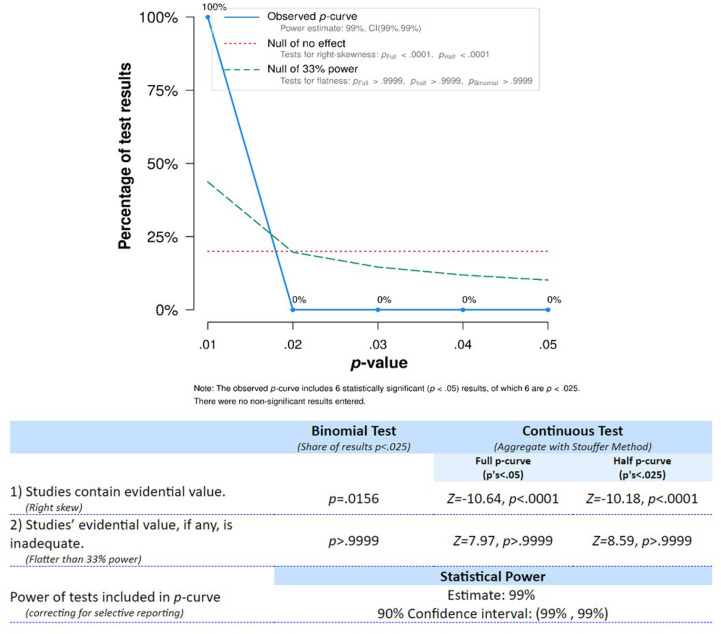
*p*-curve distribution for tests studying the association between finger gnosis and numerical abilities.

As evidenced by the right-skewed distribution of the *p*-curve (see Figure 4), the tests entered into the analysis were considered to provide evidential value and had high statistical power. Therefore, the association of finger gnosis and numerical skills seems to have evidential value and should continue to be investigated for further clarification of underlying mechanisms.

In sum, while recent evidence endorsed the idea that finger gnosis may uniquely predict the development of numerical competences (see Figure 2), it also suggests that its impact may be less conspicuous than thought initially. Although this seems to speak against the claim that well-developed finger gnosis at an early age may be an important advantage for future numerical development, the questions of why and how this association emerges (and yet, is repeatedly evidenced) remains unanswered. In this context, considering the influence of FMS on numerical development might be informative to endorse or refute a functional explanation of these findings.

### Fine Motor Skills and Numerical Abilities

Most studies investigating the association between FMS and numerical competences relied on a rather general construct of FMS. For instance, considering six sets of large-scale longitudinal data, Grissmer et al. (2010) found that FMS assessed in kindergarten were a better predictor of later mathematics achievement than measures of attention (see Table 2). Similarly, Luo et al. (2007) found that FMS significantly predicted mathematics achievement at kindergarten entry even after partialling out influences of other background variables such as sex, age, and socioeconomic status (see also Son and Meisels, 2006; Pagani et al., 2010).

**TABLE 2:** Overview of studies examining the association between fine motor skills and numerical skills.

**References**	**Finger variable**	**Age (Y;M)**	**N**	**Task**	**Control variables**	**Numerical outcome variables^1^ (with reported effect sizes)**
Asakawa and Sugimura (2014)**^L^**	Finger dexterity (FD) (T1, T2, T3, and T4)	T1: 4;8 T2: 5;2 T3: 5;8 T4: 6;2	33	Pegboard	Age, gender, rhythmic hand movement (T1, T2, T3, and T4)	**Ca:** addition *FD T1 r* = 0.53 (T1), *r* = 0.34 (T2), *r* = 0.48 (T3), *r* = 0.36 (T4) *FD T2* ***r* = 0.58** (T2), ***r* = 0.44** (T3), ***r* = 0.32** (T4) *FD T3* ***r* = 0.57** (T3), ***r* = 0.38** (T4) *FD T4* ***r* = 0.55** (T4)
Cameron et al. (2012)**^L^**	Fine motor skills (T1)	T1: 5;0 T2: 5;4 T3: 5;9	213	Block building, copying, drawing	Gender, ethnicity, age, maternal education, executive function, gross motor skills (T1)	**Ca:** applied problems fine motor composite – *r* = 0.17 (T2), *r* = 0.25 (T3) blocks – *r* = 0.11 (T2), *r* = 0.17 (T3) design copy – *r* = 0.16 (T2), *r* = 0.24 (T3) draw-a-person – *r* = 0.10 (T2), *r* = 0.08 (T3)
Dinehart and Manfra (2013)**^L2^**	Fine motor object Manipulation (FMOM) and Fine motor writing (FMW) (T1)	T1: 5;2 T2: ∼8;2	3234	Block building, string weaving, bead stringing; page turning, pegboard; cutting; play dough; paper folding	Expressive & receptive language, matching, counting (T1), gender, ethnicity, SES, school absences	**Math achievement** (**Ca, T2)**: FMOM: *r* = .21, Cohen’s *d* = .14 (GPA), *r* = .22, *d* = .09 (SAT10) FMW: *r* = 0.31, Cohen’s *d* = 0.21 (GPA), *r* = 0.33, *d* = 0.11 (SAT10)
Fischer et al. (2017)**^C^**	Fine motor skills	4;6	177	Pegboard, bead-threading, block turning	General cognitive ability, age, home math, home FMS	**Co:** procedural counting ***r* = 0.41**, β = 0.31 Conceptual counting ***r* = 0.36**, β = 0.21 (total effect)
Gashaj et al. (2018)**^L^**	Fine motor skills	T1: 6;5 T2: 8;0	136	Bead-threading, coin posting, drawing within boundaries	Numerical skills, executive functions	**M, N:** magnitude comparison (S), number line estimation (S & NS) **β = 0.31** (concurrent) **Math achievement (N, Ca):** β = 0.09 (longitudinal)
Gashaj et al. (2019)**^C^**	Fine motor skills	6;5	151	Bead-threading, coin posting, drawing within boundaries	Numerical skills, executive functions (regression models), age (correlations)	**M, N:** magnitude comparison (NS) *r* = 0.15, β = 0.14 Magnitude comparison (S) *r* = 0.22, β = 0.09 Number line estimation (NS) ***r* = 0.42**, β = 0.33 Number line estimation (S) ***r* = 0.36**, β = 0.02
Grissmer et al. (2010)**^L2^**	Fine motor skills (T1)	T1: ∼5;0 T2: ∼6;0^3^	21.260 (ECLS-K) 2714 (NLSY) 11.200 (BCS)	Block building, design copying, drawing	Social skills, attention, gross motor skills, early math, early reading	**Math achievement (N, Ca)**: FMS: β = 0.14 (ECLS-K, T2) Motor/social: β = 0.05 (NLSY, T2) Copying: β = 0.36, Drawing:.09 (BCS, T2)
Kim et al. (2017)**^L4^**	Fine motor coordination (FMC) and visuomotor integration (VMI) (T1, T2, and T3)	T1: 5;6 (beginning KG) T2: end KG^3^ T3: end 1^st^ grade	135	Design copying, speeded drawing within boundaries	Age, gender, SES, treatment condition	**Ca, M**: mathematics skills (T1, T2, T3) *FMC (T1)*: *r* = 0.24, *r* = 0.23, *r* = 0.21 *FMC (T2)*: *r* = 0.18, *r* = 0.14, *r* = 0.03 *FMC (T3): r* = 0.24, *r* = 0.16, *r* = 0.15/β = 0.33 *VMI (T1):* ***r* = 0.57**/β = 0.43, ***r* = 0.61**/β = 0.13, ***r* = 0.58** *VMI (T2)*: ***r* = 0.53**, ***r* = 0.59**, ***r* = 0.58**/β = 0.14 *VMI (T3)*: ***r* = 0.54**, ***r* = 0.56**, ***r* = 0.67**
Luo et al. (2007)**^L2^**	Fine motor skills (T1)	T1: 5;7 T2: 6;2 T3: 7;2	10060 9816 EUA^5^ 244 EAA^6^	Block building, design copying, drawing	Gender, age, mother’s and father’s education, SES, parental educational expectations	**Math achievement growth rate** - **Co, N, Ca** (T1, T2, and T3): *B* = 1.68 (intercept) *B* = 0.09 (slope)
Pagani et al. (2010)**^L2^**	Fine motor skills (T1)	T1: 5;5 T2: ∼ 7,5	1,145	Object manipulation	Early math and reading, age, gender, ethnicity, health, birth time and weight, SES (T1)	**Math achievement** (teacher-reported): ***r* = 0.30** (T2)
Penner-Wilger et al. (2007)**^C^**	Finger agility	6;10	146	Finger tapping	Gender, receptive vocabulary, processing speed	**N**: *r* = .18 **Ca**: *r* = .12
Pitchford et al. (2016)**^C^**	Fine motor precision (FMP) and fine Motor integration (FMI)	Study 1: 5;5 – 6;8 Study 2: 4;0 – 6;0^3^	Study 1: 62 Study 2: 34	Design copying, drawing, folding and cutting within boundaries	SES, gender, verbal and nonverbal IQ, verbal STM (Studies 1 and 2)	**Math achievement** – **Ca**: *Study 1* FMP: ***r* = 0.60/**β = 0.42 FMI: ***r* = 0.57**/β = 0.16 *Study 2* FMP: ***r* = 0.31** FMI: ***r* = 0.50**
Son and Meisels (2006)**^L2^**	Fine motor skills (T1)	T1: 5;5 T2: ∼6;11	12,583	Block building, design copying, drawing	Achievement in T1, age, gender, ethnicity, SES	**Math Achievement** – **Co, N. Ca**:***r* = 0.44** (T1), ***r* = 0.48** (T2)
Suggate et al. (2017a)**^C^**	Fine motor skills	4;9	81	Pegboard, bead-threading, block turning	Age, receptive vocabulary	**Co, Ca:** numerical skills (total): *r* = 0.73/β = 0.34 finger numerical skills: *r* = 0.69/β = 0.40 non-finger numerical skills: *r* = 0.70/β = 0.24

*Medium to large effect sizes (Cohen, 1988) are displayed in bold typeface. Regression coefficients were not interpreted in terms of effect size. ∼Indicates an approximate mean age when exact means are not provided. Tests included in the *p*-curve analysis are underlined. L, longitudinal; C, cross-sectional. ^1^In order to facilitate comparisons between studies, we grouped the outcome variables into four different categories whenever possible (Wyschkon et al., 2015): Co, counting (i.e., forward, backward, from *x* to *y*); N, number (e.g., number reading, symbol-magnitude mapping, cardinality, and place value); Ca, calculation (e.g., addition and subtraction); M, magnitude (e.g., subitizing, size comparison, and magnitude judgment). Original outcome measure names as described in the studies are cited when different from category names. ^2^Studies based on large scale assessment data sets. The Early Childhood Longitudinal Study (ECLS-K) was used by Son and Meisels (2006); Luo et al. (2007), and Grissmer et al. (2010). Additionally, the National Longitudinal Survey of Youth (NLSY) and the British Cohort Study (BCS) were also used by Grissmer et al. (2010). Lastly, the Miami-Dade School Readiness Project (M-DSRP) was used by Dinehart and Manfra (2013) and the Quebec Longitudinal Study of Child Development (QLSCD) by Pagani et al. (2010). ^3^It was not possible to retrieve information regarding mean age at assessment time points. ^4^The study also included a 1 ^*st*^–2^*nd*^ grade cohort which was not considered for the present review. Only the Kindergarten (KG) cohort was included. ^5^European American children. ^6^East Asian American children.*

However, as these studies derived a single FMS score based on performance on drawing, copying and block building tasks (see Table 2), they lacked differentiation between specific subcomponents which might contribute specifically and differentially to the development of numerical skills. More recent studies aimed at filling this gap. Pitchford et al. (2016), for instance, examined the specific contribution of two types of FMS distinguishable by how much they rely on visual-perceptual processing, namely (a) fine motor integration (which requires coordinated hand-eye movements and visual-perceptual integration for adequate motor output) and (b) fine motor precision (a more pure measure of FMS indexed by tasks of drawing, folding and cutting within given boundaries). Performance on visual-perceptual integration tasks administered in first grade was found to be a better predictor of concurrent mathematics achievement than of reading ability, even after accounting for influences of general cognitive ability (see Figure 2).

An alternative characterization of FMS was suggested by Dinehart and Manfra (2013), who proposed the existence of two highly correlated but distinct subcomponents of FMS: (a) fine motor object manipulation, which requires manual dexterity and is necessary for placing pegs in holes, lacing, and building with blocks; and (b) fine motor writing (i.e., graphomotor skills), a more complex ability which requires several cognitive and neuromotor processes and is necessary for drawing or writing. The authors found that both fine motor object manipulation and fine motor writing skills assessed in kindergarten exerted unique influences on second grade mathematics scores (see Table 2), with a larger effect size for fine motor writing (see also Cameron et al., 2012). Similar results were found by Kim et al. (2017), who found that preschoolers’ visuomotor integration performance was associated with their numerical skills measured at the end of first grade.

In order to isolate FMS from contamination by visual-spatial skills, Penner-Wilger et al. (2007) used a computerized version of a finger tapping task and found that finger agility contributed directly and uniquely to the concurrent prediction of first graders’ number system knowledge, but not calculation skills. Asakawa and Sugimura (2014) also investigated the relationship between FMS and numerical skills more differentially and found that finger dexterity predicted participants’ arithmetic performance more strongly than it predicted their vocabulary skills. Additionally, these authors observed that the association between FMS and numerical skills was already strong in 4-year-old children, suggesting that the relation between finger dexterity and numerical skills emerges very early in life.

More recently, Gashaj et al. (2019) examined the concurrent and longitudinal (Gashaj et al., 2018) associations of FMS (as measured by bead threading, coin posting and drawing within boundaries at age 6), executive functioning and numerical abilities. After accounting for the influence of numerical precursor skills and executive functions, the authors observed that FMS significantly predicted non-symbolic (but not symbolic) number line estimation in 6-year-old children (Gashaj et al., 2019). However, using structural equation modeling, they found that FMS at age 6 only predicted mathematics achievement in second grade indirectly through basic numerical abilities such as magnitude comparison and number line estimation, but not directly (Gashaj et al., 2018).

The evidential value of these findings was also evaluated by means of a *p*-curve analysis (Simonsohn et al., 2014, 2015). All included tests were selected based on the same criteria previously used for selection of the finger gnosis findings (see Table 2 for disclosure) with two new added criteria: (1) when multiple FMS scores were given (e.g., Cameron et al., 2012), we selected either the more comprehensive score or the one mirroring our operational definition of FMS (e.g., Kim et al., 2017); (2) when tests for multiple numerical dependent variables were provided, we opted for the one with the highest predictive value (expressed by its beta weight in a regression analysis; Suggate et al., 2017a) or largest effect size (expressed by Cohen’s *d*; Dinehart and Manfra, 2013).

**FIGURE 5:**
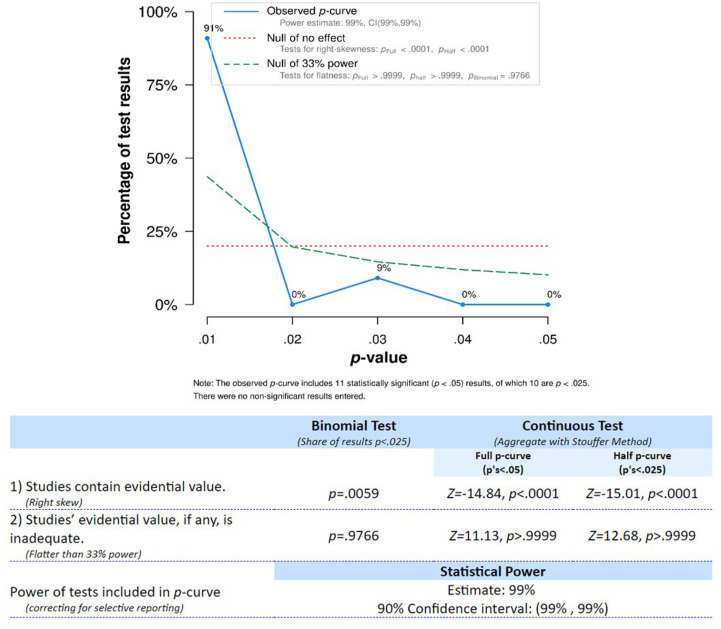
*p*-curve distribution for tests studying the association between fine motor skills and numerical abilities.

As expected from the large sample sizes of nearly all included studies, the evidential value of these findings was corroborated by a right-skewed distribution of the *p*-curve (see Figure 5) with again high statistical power. Therefore, the validity of the association of FMS and numerical skills is corroborated and thus merits further investigation.

Taken together, these studies point to a clear contribution of FMS to numerical and mathematical abilities, most specifically in what regards mathematics achievement but also number system knowledge and arithmetic abilities (see Figure 1). Importantly, however, there appear to be subtle differences across studies in what is subsumed under the term FMS as well as some terminological disagreement among researchers. For instance, while some authors use the terms “manual dexterity” and “FMS” interchangeably as having the same meaning (Makofske, 2011), others consider the first to be a specific subtype of FMS (Houwen et al., 2008). Additionally, most studies investigating FMS so far used a composite measure of different subcomponents, including tasks heavily based on visual-motor skills (e.g., Son and Meisels, 2006; Luo et al., 2007; Grissmer et al., 2010; see Table 2). To the best of our knowledge, the few existing studies which attempted to isolate contributions of different aspects of FMS to numerical abilities (Dinehart and Manfra, 2013; Pitchford et al., 2016) still lacked an effective dissociation of a type of fine motor ability which is goal-oriented and visually guided from a second type which consists of the mere motor act of controlling and coordinating finger movements. This distinction may be crucial for understanding the relevance of FMS for numerical development because the first type involves many other (cognitive) processes, such as visual-spatial skills and components of executive function such as planning and inhibition. Recent studies (Gashaj et al., 2018, 2019) tackled this issue by controlling for influences of executive functioning, which may be a further promising direction for disentangling influences of FMS from those of visual-spatial skills in addition to indexing FMS by finger tapping performance (Penner-Wilger et al., 2007). Nevertheless, further studies are needed to further delineate specific connections between finger motor skills and numerical abilities.

### Finger Gnosis, Fine Motor Skills, and Finger-Based Numerical Strategies

Although finger gnosis and FMS seem to make specific contributions to the development of numeracy (Penner-Wilger et al., 2007, see Figure 1), it is possible that they reflect different dimensions of finger-based numerical strategies which may be dissociable and stem from different mechanisms. To this date, only one study attempted to disentangle the specific contributions of finger gnosis and FMS (Penner-Wilger et al., 2007) to the development of numerical skills. Results showed that, while finger gnosis seemed to be associated with both number system knowledge and calculation skills, FMS (in this study, finger agility) were only found to relate to number system knowledge. The authors chose finger agility as a proxy for FMS due to its relative independence from visual-motor integration skills, which may be considered a confound. These initial findings hint at the need to further investigate the specific contributions of different FMS components and finger gnosis to the development of numerical skills.

From a functional perspective, it is nonetheless easy to fathom how finger gnosis and FMS may be intertwined. For instance, to effectively count on one’s fingers, one must be able to recognize them as separate entities and assign different numerical magnitudes to each finger while moving them individually. Thus, the success in using one’s fingers to count relies both on good differentiability and adequate movement capacity of fingers. In this line of thought, the existence of a functional relation between both finger gnosis or FMS and numerical abilities may be corroborated. In functionalist proposition Butterworth (1999), fingers and numbers are indirectly related through children’s use of their fingers to represent quantities, extending number processing beyond the subitizing range and serving as functional aids in numerical representation and computation. The role of fingers then would be that of a “missing tool” for the connection of non-symbolic and symbolic number representations which are necessary for numerical computations (Andres et al., 2008, see also Gallistel and Gelman, 1992).

Recent evidence provides further support for this claim. For the case of FMS, Fischer and colleagues (2017) found that the association between FMS (as measured by bead-threading, block turning, and a pegboard task) and conceptual counting knowledge in preschool children was mediated by procedural counting knowledge. This finding suggests that children with better FMS may be more successful at using their fingers for counting procedures, which might in turn facilitate the acquisition of a conceptual understanding of counting.

Similarly, Suggate et al. (2017a) found that preschoolers’ FMS (indexed by bead-threading, block turning, and a pegboard task) were more strongly related to performance in counting and arithmetic tasks that involved the use of finger-based strategies than to those tasks that were solved without help of fingers, even after controlling for the influence of age, vocabulary, and general cognitive ability. Moreover, the association of FMS and non-finger-based numerical tasks was entirely mediated by finger-based numerical skills, supporting the idea of finger-based strategies as a link between FMS and numerical development.

Moreover, for the case of finger gnosis, Reeve and Humberstone (2011) found that preschool children’s finger gnosis was related to whether they used their fingers while performing calculations as well as to their performance in a calculation task. In particular, children with poor finger gnosis barely used their fingers and committed more errors while calculating. Furthermore, Costa et al. (2011) observed that dyscalculic children had significantly poorer finger gnosis, even though their general cognitive ability and working memory were at typical level. In their study, finger gnosis was particularly relevant for solving word problems, which required manipulations of quantities between 1 and 10, for which the use of fingers may be specifically suited. The authors argued that finger gnosis deficits relate to an inability to use fingers to transiently represent magnitudes. Furthermore, a recent study by van Rinsveld et al. (2020) found that preschoolers’ performance in a finger pattern recognition task was a better longitudinal predictor of their number line estimation performance at the beginning of first grade than finger gnosis. In particular, the authors observed that, although finger pattern recognition was concurrently correlated with finger gnosis, only the former predicted children’s later number line estimation. These findings seem to corroborate the idea of a rather indirect role of finger gnosis for the acquisition of number representations in that it may scaffold the emergence of finger-based numerical representations. In sum, this evidence supports the assumption that the association between fingers and numbers may be functional and stem from the usage of fingers for numerical tasks.

Nevertheless, it must be noted that the functionalist proposition is based to a large extent on the behavioral and ethnocultural evidence available at the time of its publication (Butterworth, 1999). In the meantime, neuroimaging methods saw significant improvements and a leap in popularity, giving rise to several neurofunctional and neurostimulation studies capable of specifying the neural correlates of finger gnosis and numerical abilities in more detail. This new evidence provides further insights into how the neural circuits supporting finger and number representations are intertwined. As mentioned above, overlapping activation of cortical networks for number processing and finger movement can be observed in children as young as 8 years old and is still observed in adulthood (e.g., Kaufmann et al., 2008), when fingers are most likely no longer used in aid of numerical processes.

Although this observation speaks in favor of a common neural substrate for representing numbers and fingers, it does not provide clarifying information on the origins of this shared neural circuitry. In lieu of a functionalist explanation, it is in principle likewise possible that the neural circuitry supporting sensorimotor finger function is also at least partially involved in number representation and numerical operations through evolutionarily redeployment mechanisms. Although the massive redeployment hypothesis does not preclude that numerical representations may be in some way grounded in sensorimotor experience (Anderson, 2010), early finger usage is thought to be no more than a useful tool for physically (and spatio-temporally) representing to-be-learned concepts with no semantic grounding resulting from these actions. That is not to say that finger-based strategies are not a useful resource for numerical learning, but rather that their application may be analogous to the purpose of speech-accompanying gesturing, that is, an outlet for conveying ideas not yet suited for verbal expression (Goldin-Meadow, 2003; Anderson, 2010).Finally, it should be noted that the association of fingers and numbers on the neural level may be accounted for by another interpretation, namely, the neuronal recycling hypothesis (e.g., Dehaene, 2009). Although the neuronal recycling hypothesis appears similar to the massive redeployment hypothesis, they differ in their definition of how exactly fingers and numbers come to be served by common neural circuits: while the neuronal recycling perspective posits that this may be the product of learning-driven neuronal plasticity (Dehaene, 2009), the massive redeployment hypothesis pins down the origin of this association to human phylogenesis. In other words, while the first assumes the association of fingers and numbers to be the product of human development, the second attributes it to the repurposing of phylogenetically older neural systems to support evolutionarily recent functions such as numerical reasoning.

While the neuronal recycling account may complement the functionalist hypothesis where the latter does not delve into detail – that is, the neurofunctional network sustaining the finger-number association – assuming a complete independence of these systems prior to the onset of developmental experience may be hasty. After all, as argued by Jones (2018), neural plasticity is a process which may be too slow-paced to satisfactorily explain how neural systems supporting number processing may shift so rapidly in function. Therefore, experiential events connecting fingers to numbers may serve the purpose of increasing connectivity between the respective neural systems, which may already have been associated to some extent to begin with. Yet, on the other hand, it is widely known that learning may lead to considerable changes in functional but also structural aspects of the brain. In the end, the most likely scenario is that all explanatory accounts on the behavioral and neuro-functional association of fingers and numbers may be at least partially correct but also partially incorrect. That is to say that, while there may be an innate disposition for numerical abilities to be grounded in the sensorimotor systems subserving fingers, certain developmental experiences would still be required for said association to unfold.

Although this claim seems to be supported by both behavioral as well as neurophysiological data, further studies are necessary to disentangle the nature vs nurture mechanisms of the association of finger and numbers. Exploring these associations before the onset of “nurture” influences – that is, before children start using their fingers for counting and representing numerical magnitudes – may be one promising direction for disentangling these explanations. To this end, it may also be informative to explore differential neural activation for finger gnosis and FMS, as they may reflect different aspects or degrees of functionality of associations between fingers and numbers. Finally, some additional insights on the innateness of a shared neural circuitry for fingers and numbers may be gained from animal (e.g., Shoham and Grinvald, 2001) or computational models (e.g., De La Cruz et al., 2014; Di Nuovo and McClelland, 2019). Further neurofunctional or electrophysiological studies of people belonging to cultures with non-finger-exclusive embodied counting systems or with a limited to non-existent representational system for exact number (e.g., Pica et al., 2004; Frank et al., 2008) may also be particularly enlightening for elucidating the causality direction of this association.

## Conclusion and Perspectives

Taken together, the studies reviewed above seem to point to a specific and unique contribution of finger-related variables to the development of numerical skills that seems to persist over and above the influence of other important predictors such as general cognitive ability or numerical precursor skills. In particular, finger gnosis and/or FMS were observed to predict measures of counting (Fayol et al., 1998; Noël, 2005; Luo et al., 2007; Penner-Wilger et al., 2009; Poltz et al., 2015), number system knowledge (Fayol et al., 1998; Noël, 2005; Son and Meisels, 2006; Luo et al., 2007; Penner-Wilger et al., 2007, 2009; Poltz et al., 2015), number magnitude processing (Noël, 2005; Son and Meisels, 2006; Luo et al., 2007; Poltz et al., 2015), and calculation ability (Fayol et al., 1998; Noël, 2005; Son and Meisels, 2006; Luo et al., 2007; Penner-Wilger et al., 2007; Dinehart and Manfra, 2013; Asakawa and Sugimura, 2014; Poltz et al., 2015; Long et al., 2016; Pitchford et al., 2016; Wasner et al., 2016). Furthermore, finger gnosis and FMS were found to be better predictors of some numerical outcome measures than of other variables such as reading ability (Noël, 2005) and vocabulary (Asakawa and Sugimura, 2014).

However, the contribution of both finger gnosis and FMS to numerical development seems to be smaller than previously thought with, for instance, finger gnosis explaining about 1–2% of variance of first graders’ calculation skills (e.g., Wasner et al., 2016) after controlling for domain-general skills as well as natural variables such as general cognitive ability and age. Although results from training studies of both FMS (Atsushi et al., 2017) and finger gnosis (Gracia-Bafalluy and Noël, 2008; but see Fischer, 2010 for methodological limitations as well as Jay and Betenson, 2017 for differing results) showed improvements on first graders’ basic numerical and arithmetical skills, the longitudinal evidence presented above is hard to reconcile with the idea of finger gnosis and/or FMS being necessary component skills of numerical abilities. However, this does not imply that finger-related variables are not relevant for children’s numerical development. As suggested by recent evidence, finger gnosis and FMS may be functionally relevant for the acquisition of numerical skills in that they support the successful use of finger-based numerical strategies such as finger counting or calculating (Reeve and Humberstone, 2011; Fischer et al., 2017; Suggate et al., 2017a, but see Lafay et al., 2013).

In line with this, Roesch and Moeller (2015) recently discussed the influence of finger-based numerical strategies in the light of a current model of early numerical development (Krajewski and Schneider, 2009). They argued that fingers do not only help children in counting, reciting number words and grasping the concept of cardinality (for similar conclusions, see also Gunderson et al., 2015), but also serve as a tool for corroborating initial arithmetic operations such as part-whole relations. As such, finger-based numerical strategies may support early numerical development at all stages specified by Krajewski and Schneider (2009) (see also Figure 1) as well as bolster the acquisition of foundational numerical abilities described by influential authors in the field of numerical development (e.g., Gelman and Gallistel, 1978; Butterworth, 1999; Geary, 2007).

Therefore, although using fingers may not be imperative for the acquisition of basic numerical concepts (Nicoladis et al., 2010; Crollen et al., 2011), finger-based strategies constitute a natural scaffold for the development of crucial numerical abilities and may be highly advantageous for most – if not for all – children in early stages of their numerical development. This may be further evidenced by studies specifically designed to detect differences in specific numerical abilities which may be more directly supported by use of fingers, as well as expanding the examined age range to even younger children in order to capture developmental windows in which finger-related abilities may more directly influence the acquisition of numerical skills. Specifically, evaluating whether and if so, how FMS mediate the association between finger gnosis and numerical abilities may be crucial to unraveling the causality controversy. Furthermore, when examining the associations of finger sensory and motor abilities, finger-based strategies and numerical abilities, it would be desirable to investigate not only whether children use their fingers for numerical computations, but how they do so. This may be relevant because finger-based strategies may vary in terms of efficiency and complexity (Björklund et al., 2019) both from a cognitive and from a motor perspective, potentially leading to differential associations between finger sensorimotor skills and numerical outcomes.

Moreover, even if it seems plausible to conclude that higher finger gnosis and FMS may lead to more successful finger usage for counting and initial calculation, it might be that they constitute a consequence rather than a cause of frequent and differential finger use for number processing. In line with this, Poltz et al. (2015) observed a bi-directional relation between finger gnosis and numerical development, as not only children’s numerical ability was longitudinally predicted by finger gnosis, but also finger gnosis was predicted by earlier numerical performance – even though the second association was weaker.

Furthermore, cross-sectional and correlational evidence do not suffice for pinpointing the mechanisms precipitating the association of fingers and numerical representations. As such, the existing evidence may not be sufficient to fully endorse either the functionalist or redeployment explanation of empirical findings. Crucially, the latter regards this association as innate rather than functionally acquired, arguing that the natural inclination to use fingers for representing numerical quantities feels natural because the neural circuits supporting finger motor and sensory skills have been redeployed for supporting numerical representations (Penner-Wilger and Anderson, 2013). In fact, Anderson (2010) argued that “the motor control system is here [for representing numerical information] being used for a specific cognitive purpose not because it is performing semantic grounding or providing metaphorically guided domain structuring, but because it offers an appropriate physical (and spatiotemporal) resource for the task” at hand (p. 256).

However, observed cross-cultural differences in embodied (finger) counting systems appear to reflect the existence of functional mechanisms influencing the association of fingers and numbers to some extent. For instance, although finger counting seems to be culturally universal, non-finger-based embodied strategies were found to be part of the counting system of the new Guinean Oksapmin community (Butterworth, 1999; Ifrah, 2000; Saxe and Esmonde, 2004; Bender and Beller, 2012), who employs body parts such as shoulders, eyes and nose in addition to their fingers for counting. The fact that embodied counting systems may not be entirely limited to fingers is not directly explained by redeployment mechanisms, as shared sensorimotor circuits for number processing seem to be specific to finger movements (e.g., Michaux et al., 2013).

Further evidence supporting a functional association of fingers and numbers can be found in studies employing different research methodologies. For instance, on a *behavioral* level, it was observed that a certain type of addition and subtraction errors (i.e., getting the answer to a problem wrong by ±5) can be observed in primary school-children at the time when multiplication is introduced to them (Domahs et al., 2008). These so-called split-five errors in mental calculation were interpreted to reflect a failure to account for one full-hand-unit, suggesting that finger-based strategies influence mental calculations specifically. In line with this, sequential finger movements were found to interfere only with arithmetic operations ontogenetically supported by using fingers, that is, in addition but not in multiplication (Michaux et al., 2013).

Moreover, *electrophysiological* evidence indicates that right-hand muscles were activated on a parity judgment task with small numerals (i.e., 1–4) in participants who started counting on their right hands (Andres et al., 2007), suggesting that the activation of hand motor circuits in number processing seems to be modulated by individual differences in finger counting routines (Sato et al., 2007).

Finally, in a *cross-cultural* study, Domahs et al. (2010) found that adult symbolic magnitude processing is influenced culture-specific aspects of the respective finger counting habits (i.e., finger postures for numbers from 6 to 10 require both hands in German but only one hand in Chinese finger counting routines). In particular, German participants took more time for magnitude comparisons on pairs of symbolic numbers of which at least one required a two-hand posture as compared to one-hand postures in the Chinese finger counting routine (e.g., 6 vs 9). This finding is particularly compelling because, if number processes were not considered to be somehow shaped by cultural specificities of finger-based numerical strategies, such influences of specific properties of finger counting routines should not be observed on a cross-cultural level. Furthermore, recent *developmental* findings suggesting that (culture-specific) finger-based numerical strategies (or representations) mediate the association of numerical skills with either finger gnosis or FMS (Costa et al., 2011; Reeve and Humberstone, 2011; Fischer et al., 2017; Suggate et al., 2017a; van Rinsveld et al., 2020) also corroborate a functionalist stance of associations between fingers and numbers.

Finally, on a *sensorimotor* level, numerical processing seems to be facilitated not only by posturing cardinal finger patterns (Sixtus et al., 2017) but also by ordinal aspects of finger counting, namely by tactile stimulation matching the last finger used to count to a certain number on the respective finger counting routine (Sixtus et al., 2020). Such individual and culture-specific differences may not be directly expected under the massive redeployment hypothesis, at least not in the version described by Penner-Wilger and Anderson (2013). Taken together, these research findings seem to corroborate the idea that the association of fingers and numbers is functionally modulated and may emerge from the use of finger-based numerical strategies in early stages of children’s numerical development.

However, contemplating this (mostly) behavioral evidence does not resolve the chicken-or-egg conundrum involving the functionalist vs redeployment debate, as some functionally driven variability is also to be expected (although to a lesser extent) from the massive redeployment hypothesis (Anderson, 2010). Crucially, we argue that these theoretical accounts are not mutually exclusive and may thus not necessarily need to be treated as an either-or-question. Instead, the motor behavior of finger counting might be the developmental process by which number representations are grounded in the finger sensorimotor system, which may already have a predisposition to accommodate these (Lakoff and Núñez, 2000). Therefore, once these numerical representations become developmentally connected to the finger sensorimotor circuitry, they become permanently associated both on a neurofunctional and on a behavioral level, resulting in widespread associations between fingers and numbers.

Although this proposition seems to render a plausible explanation for associations between fingers and numbers, it needs to be substantiated by future studies. In order to expand on the topic, some research directions may be particularly fruitful. First, on a behavioral level, further longitudinal or training studies may disentangle the relations between early finger gnosis and FMS, finger-based numerical strategies, and early numerical abilities while controlling for the influence of domain-general variables. Moreover, contemplating individual differences in the association of finger gnosis, FMS and numerical abilities may inform why and to what extent some children might prefer and benefit more from strategies other than finger usage. Additionally, future research should aim at establishing a gold standard for measuring finger gnosis as well as increase the reliability of finger gnosis tasks to avoid confounds and ensure comparability between studies. Second, on a neurofunctional level, it may be informative to investigate the neural circuitry subserving numerical representations and finger movements prior to the adoption of functional strategies, as well as explore differential activations for finger gnosis and fine motor ability. Finally, some additional insights into the innateness of a shared neural circuitry for fingers and numbers may be gained from cross-cultural studies, as well as animal and computational modeling. Disclosing the driving mechanisms of the association between finger sensorimotor skills and early numerical development would represent a breakthrough to both psychological and mathematics education research, as it may help establish a common ground on the potentials but also limitations of finger-based numerical strategies for educational practice.

## Author Contributions

All authors contributed meaningfully to the preparation of this manuscript. RB was responsible for the conception and design of the work, literature search, writing up the manuscript, and designing tables and figure. SR was responsible for the conception of the work, writing up parts of the discussion and conclusion, and for critical revision. CG offered critical revision through all stages of the manuscript preparation. KM contributed to the theoretical conception and design of the work and actively revised the manuscript from the first draft to the final version. All authors approved the final version of this manuscript.

## Conflict of Interest

The authors declare that the research was conducted in the absence of any commercial or financial relationships that could be construed as a potential conflict of interest.
